# The Impact of NO_2_ on Epithelial Barrier Integrity of a Primary Cell‐Based Air–Liquid Interface Model of the Nasal Respiratory Epithelium

**DOI:** 10.1002/jat.4717

**Published:** 2024-11-12

**Authors:** Helena Moratin, Josephine Lang, Magdalena‐Sophie Picker, Angela Rossi, Christian Wilhelm, Armin von Fournier, Manuel Stöth, Miguel Goncalves, Norbert Kleinsasser, Stephan Hackenberg, Agmal Scherzad, Till Jasper Meyer

**Affiliations:** ^1^ Department of Oto‐Rhino‐Laryngology, Head and Neck Surgery University Hospital Würzburg Würzburg Germany; ^2^ Translational Center Regenerative Therapies (TLC‐RT) Fraunhofer Institute for Silicate Research (ISC) Würzburg Germany

**Keywords:** hypoxia, nasal epithelial barrier, NO_2_, tight junctions, upper airway

## Abstract

Nitrogen dioxide (NO_2_) is a pervasive gaseous air pollutant with well‐documented hazardous effects on health, necessitating precise toxicological characterization. While prior research has primarily focused on lower airway structures, the upper airways, serving as the first line of defense against airborne substances, remain understudied. This study aimed to investigate the functional effects of NO_2_ exposure alone or in combination with hypoxia as a secondary stimulus on nasal epithelium and elucidate its molecular mechanisms because hypoxia is considered a pathophysiological factor in the onset and persistence of chronic rhinosinusitis, a disease of the upper airways. Air–liquid interface cell cultures derived from primary nasal mucosa cells were utilized as an in vitro model, offering a high in vitro–in vivo correlation. Our findings demonstrate that NO_2_ exposure induces malfunction of the epithelial barrier, as evidenced by decreased transepithelial electrical resistance and increased fluorescein isothiocyanate (FITC)‐dextran permeability. mRNA expression analysis revealed a significant increase in IL‐6 and IL‐8 expressions following NO_2_. Reduced mRNA expression of the tight junction component occludin was identified as a structural correlate of the damaged epithelial barrier. Notably, hypoxic conditions alone did not alter epithelial barrier integrity. These findings provide information on the harmful effects of NO_2_ exposure on the human nasal epithelium, including compromised barrier integrity and induction of inflammatory responses. Overall, this study contributes to our understanding of pathophysiological mechanisms underlying also upper airway respiratory diseases associated with air pollution exposure and emphasizes the importance of mitigating NO_2_ emissions to safeguard respiratory health.

## Abstract 2

Nitrogen dioxide (NO_2_) is an air pollutant with known health risks. This study explored the impact of NO_2_ and hypoxia on nasal epithelium using air–liquid interface cultures from primary nasal mucosa cells. Results showed that NO_2_ exposure compromised the epithelial barrier, increased IL‐6 and IL‐8 expressions, and reduced mRNA expression of occludin. These findings highlight the detrimental effects of NO_2_ on nasal epithelium and stress the need for emission control.

## 1 Introduction

The nasal epithelium, acting as a crucial interface between the external environment—including air pollutants—and the respiratory system, holds a pivotal role in safeguarding the entire respiratory tract. Nasal epithelial cells (NEC) serve as key mediators in the initial defense against airborne pollutants and inhaled pathogens. Through the secretion of antimicrobial substances such as lactoferrin, lysozyme, and defensins α and β, they effectively hinder the penetration of pathogenic germs (Yang and Oppenheim 2004). Furthermore, NEC orchestrate mucosal immunity by producing functional molecules like pro‐inflammatory cytokines, chemokines, and growth factors (Toppila‐Salmi et al. 2015). The sinunasal epithelium comprises various cell types, including goblet cells, basal cells, ciliated cells, and nonciliated columnar cells, working in tandem to establish an intact barrier and facilitate mucociliary clearance (Wang et al. 2015). Goblet cells play an important role in this process by secreting mucus, which entraps pathogens and particles. Subsequently, the directed movement of cilia propels the mucus from the nasal cavity towards the esophagus, where it is transported to the digestive tract by swallowing (Ha and Cho 2023).

Apical junctional complexes (AJC) connect epithelial cells to one another and consist of tight junctions (TJ), adherens junctions, desmosomes, and hemidesmosomes, with TJ being the most apically located component. The task of AJC is to form an intact barrier that regulates the paracellular passage of substances and maintains the homeostasis and polarity of the cellular network (Guttman and Finlay 2009). Malfunction of the epithelial barrier has been attributed to reduced expression of TJ‐associated proteins in vivo. For example, the presence of occludin, a key molecule of TJ, is reduced in patients with allergic rhinitis (AR) compared to healthy controls (Wang et al. 2021). The prevalence of allergic diseases has demonstrably increased in recent decades, and one hypothesis suggests causality between the increase and an elevation in airborne pollutants (Platts‐Mills 2015). To investigate this idea, various in vitro studies and investigations on mouse models have been carried out. Diesel exhaust particles and fine particulate matter ≤ 2.5 μm have been shown to induce TJ disruption and aggravate AR‐associated symptoms (Fukuoka et al. 2016; Kim et al. 2019). Exposure to ozone gas also damaged the epithelial barrier in mice, as evidenced by a reduction in TJ proteins (Lu et al. 2024). In addition to triggering allergic symptoms, environmental factors can harm the cardiovascular system and affect the entire respiratory tract. The socio‐economic impacts and effects on the quality of life of those affected by AR or chronic rhinosinusitis (CRS), which often present as comorbid conditions, are therefore severe over time (Fokkens et al. 2023; Helman et al. 2020). A clear understanding of the pathophysiological effects of exposure to environmental pollutants is essential for well‐balanced risk management.

This study focuses on nitrogen dioxide (NO_2_), which results from the reaction of nitrogen monoxide (NO) with ozone (O_3_). Combustion processes using fossil fuels, such as those in fuel engines or the burning of coal or wood, are sources of NO (Frampton et al. 1989). The primary sources of NO_2_ emissions are combustion from the energy industry and the transport sector, particularly from diesel vehicles. To a lesser extent, emissions from private households, such as those from gas stoves, cigarette smoke, or agriculture, contribute, too. Due to regular media reports, there is a high level of public attention regarding air pollution in urban areas especially in Germany. Despite eco‐political regulations, the determined limit values for airborne pollutants are regularly exceeded, particularly in densely populated inner‐city sectors (Hoffmann et al. 2022).

There are already well‐established connections between the occurrence of a broad spectrum of diseases and exposure to NO_2_. The literature reports a statistically verified association between NO_2_ exposure and childhood asthma, incidence and mortality of chronic obstructive pulmonary disease (COPD), and the incidence of lung cancer (Achakulwisut et al. 2019; Zhang, Wang, and Lu 2018). Furthermore, there is evidence that the risk of premature birth and diabetes mellitus is increased upon exposure to NO_2_. The molecular mechanism underlying this epidemiological observation is presumably that NO_2_ is operating as a pro‐inflammatory stimulus, which causes chronic inflammation of the airways and metabolic imbalance (Saltiel and Olefsky 2017). Moreover, oxidative stress mechanisms are induced, which can cause DNA damage, as already shown in NECs (Koehler et al. 2013). Although the nasal mucosa is the primary point of contact for airborne pollutants, the majority of published studies on the effects of NO_2_ were carried out on target structures in the lower respiratory tract. For example, Persinger et al. referred to the constant inflammatory injury and consecutive repair process induced by NO_2_ in lungs that may cause airway remodeling, including development of pulmonary fibrosis (Persinger et al. 2002). Inflammatory processes are mediated by factors such as Interleukin (IL‐)6, IL‐8, tumor necrosis factor‐α (TNF‐α), interferon‐γ, and IL‐1β, and a pre‐existing inflammatory condition has been shown to aggravate effects of NO_2_ (Ayyagari, Januszkiewicz, and Nath 2004).

Previous studies have also shown an increase in inflammatory tissue reaction caused by hypoxia mediated by increased expression of, e.g., IL‐8 in the respiratory system (Philippe et al. 2015). Mucosal hypoxia is considered to be a key feature of CRS. Chronic tissue inflammation in CRS with consecutive mucosal edema can create a hypoxic environment within the sinuses via mucus obstruction and cellular remodeling. Consequently, hypoxia can lead to epithelial hyperpermeability and ameliorate penetration of inhaled pathogens. In addition, hypoxia influences the morphology, beat frequency, and therefore functionality of kinocilia (Song et al. 2017; Wong et al. 2023). We therefore hypothesized an interaction between simultaneous exposure of nasal mucosa to hypoxic conditions and NO_2_ possibly causing harmful synergistic effects on the tissue.

The aim of the present study was to analyze the effect of NO_2_ on the nasal epithelium, as it serves as the primary contact area within the upper respiratory tract, and more knowledge is needed in this particularly functionally interesting region of the respiratory system. The focus was therefore set on examining the integrity of the epithelial barrier. Additionally, the study addressed the effect of NO_2_ in combination with hypoxia, a known damaging factor of the epithelial barrier, which is induced through chronic tissue inflammation as in CRS. To investigate the mechanistic background of potential effects, the inflammatory mediators IL‐6 and IL‐8 and key structure of the epithelial barrier occludin were examined.

## 2 Materials and Methods

### 2.1 Isolation and Cultivation of Human Nasal Mucosa Cells

The process of human nasal mucosa cell isolation and cultivation has been previously described (Moratin et al. 2023). Mucosa samples were acquired from 10 patients that underwent functional endoscopic sinus surgery, dacryocystorhinostomy, or turbinoplasty. Before surgery, informed consent statement was given by each patient, and the Ethics Committee of the Medical Faculty of the University of Würzburg had approved the study (vote no. 116/17). Specimen from malignant or fungal diseases were explicitly excluded. The mucosa samples were rapidly delivered from the operation room to the laboratory in saline solution. Blood clots, cartilage, and bone residues were removed with sterile forceps and scalpel after rinsing with 2 mL of MEM (Minimum Essential Medium Eagle, Sigma‐Aldrich Co. St. Louis, MO, USA). Then samples were transferred to a 15 mL tube (Greiner Bio‐One GmbH, Frickenhausen, Germany) with 9 mL of MEM plus additives. About 500 mL of MEM contained 500 μL (250 μg/mL) Fungizin/Amphotericin B, 5 mL (10.000 U/mL) penicillin/streptomycin, 500 μL (50 mg/mL) Gentamicin (all chemicals Biochrom AG, Berlin, Germany), and 5 mL L‐Glutamin (200 mM) (Sigma‐Aldrich). About 100 μL of enzyme mix was added (1 mg DNase, 100 mg protease [both Sigma‐Aldrich], 10 mL 1 × phosphate buffered saline [PBS, Roche Diagnostics, Mannheim, Germany]). The following day, porous membrane inserts for 12‐well plates were prepared for better cell attachment (Corning Transwell Polyethylenterephthalat [PET] membrane inserts, 0.4  μm; 12‐mm diameter; Corning Inc., Corning, NY, USA). Inserts were coated with 300 μL collagen (Collagen A 1 mg/mL [Biochrom AG] in PBS in a 1:1 ratio) and then placed in the incubator (CO_2_ incubator series CB, Binder GmbH, Tuttlingen, Germany) for 30 min at 37°C and 5% CO_2_. After the 30 min incubation, the PBS/collagen solution was aspirated, and the membrane was rinsed with 500 μL of PBS. About 2 mL of RPMI medium were added to the mucosa samples to stop lysis. RPMI contained 1% penicillin/streptomycin, 1% 100 mM of sodium pyruvate, 1% of a 100‐fold concentration of nonessential amino acids (Biochrom AG), and 5% fetal calf serum (FCS; Linaris, Wertheim, Germany). Sterile forceps and scalpel were used for further mechanical cell isolation, and cell suspension was filtered through a sterile compress into a 50‐mL tube (Greiner Bio‐One GmbH) followed by 5 min centrifugation at 4 C° and 1000 rpm. The supernatant was removed leaving only the cell pellet. The pellet was dissolved with 10‐mL BEGM (Bronchial Epithelial Growth Medium + 1% supplement [PromoCell GmbH, Heidelberg, Germany] + 1% penicillin/streptomycin [Biochrom AG)]. About 500 μL of the prepared cell suspension in BEGM was applied apically onto the insert membrane. A 1‐mL BEGM + 1% penicillin/streptomycin + supplement was added to the basal compartment of the well. Specimens were cultivated at 37°C and 5% CO_2_ in the incubator. Medium changes were carried out every second to third day. When cells had reached 70%–80% confluence, the cell culture was switched to the air–liquid system by removing the medium apically and changing the basal medium to fresh BEGM + penicillin/streptomycin + supplements. Experiments were performed after defined periods of time after the switch to ALI as described separately for each test.

### 2.2 Exposition With NO_2_, Normoxia, and Hypoxia

Cells were exposed to NO_2_ alone or in combination with normoxia, corresponding to 21% O_2_, or hypoxia. Furthermore, hypoxic and normoxic conditions alone were tested separately. Therefore, NECs were exposed that had been cultured at ALI‐conditions for 14 days. To avoid influence of growth factors, the basal medium of the air–liquid interface (ALI) culture was changed to supplement free BEGM 24 h prior to the experiment. The setup and process of preparation of the desired NO_2_ concentration have been previously described (Koehler et al. 2016). Shortly before, exposure cells were apically washed with 500 μL of PBS and then transferred to the Vitrocell exposure chamber (Vitrocell Systems GmbH, Waldkirch, Germany) where 0.1 ppm of NO_2_ was applied with a continuous flow of 5 mL/min for a period of 1 h. During exposure, the temperature of the cells in the exposure chamber was kept constant at 37°C using a water bath with a circulation pump. After exposure, the membranes were rinsed again with 500 μL of PBS and incubated for 24 h.

For hypoxic conditions, NECs were placed in an incubator at 37°C with 5% CO_2_ and 1% O_2_. For combined exposure with 0.1 ppm of NO_2_ and hypoxia, cells were first exposed to NO_2_ as described for 1 h, rinsed with 500 μL of PBS, and then immediately transferred to the incubator for exposure to hypoxia. Together with the hypoxia‐only samples, cells were kept in the incubator for 4 h. All cells were postincubated at 37°C with 5% CO_2_ for 24 h following exposure. At the end of this 24‐h period, all models were analyzed simultaneously. For positive control, 500 μL 1% lipopolysaccharide solution (LPS, Sigma‐Aldrich) was applied for 24 h. Post exposure cells were washed with 500 μL of PBS.

### 2.3 Fluorescein Isothiocyanate (FITC)‐Dextran Assay

FITC‐Dextran Assay was carried out to measure the permeability of ALI cell cultures with increasing number of days in culture (Days 1, 4, 7, 14 after switch to ALI). Furthermore, it was used to evaluate the influence of NO_2_, hypoxia, and the combination of both on the epithelial barrier of NEC. Dextran with 4 kDa and 40 kDa coupled with fluorescent FITC was used.

For each exposure condition, three wells were prepared. Therefore, the basolateral medium was changed to 1‐mL BEGM + penicillin/streptomycin + supplements before exposure. Approximately 4 mg of FD4 or FD40 (Sigma‐Aldrich) was mixed with 2‐mL BEGM medium + 1% pen/strep + supplement in a light‐protected 15‐mL tube. About 500 μL of the sugar solution was apically applied to the membranes of the cell cultures, and the plates were incubated in the dark for 2 h. After the incubation period, 500 μL from the basolateral compartment was transferred to a light‐protected 1.5‐mL reaction tube (Safeseal Tube 1.5 mL, product number: 72.706.001, Sarstedt AG & Co. KG, Nümbrecht, Germany). About 200 μL from this solution was applied to each well of a 96‐well plate (CELLSTAR, 96 Well Cell Culture Plate, product number: 655180, Greiner Bio‐One GmbH). Analysis with a fluorescence spectrometer at an absorption wavelength of 490 nm and an emission wavelength of 525 nm (Infinite 200 PRO, TECAN Group, Männedorf, Switzerland) was performed in the Neurological Department of the University Hospital in Würzburg to quantify the FITC‐dextran concentration which had permeated the cell layer. For each sample, blanks (BEGM without supplements) and the stock solution of FD4/FD40‐BEGM were also measured.

### 2.4 Transepithelial Electrical Resistance Measurement (TEER)

In analogy to the FITC‐assay, TEER was measured in cell cultures 14 days after transfer to ALI and after exposure to NO_2_ alone or in combination with hypoxia or normoxia to evaluate the epithelial barrier integrity. About 10 nM of tert‐butyl hydroperoxide (tBHP, Sigma‐Aldrich) was used as a positive control. The cells were treated with 500 μL tBHP for 1 h and washed with PBS prior to the measurement. One well, which was only coated with collagen‐PBS without cells, was used to evaluate exclusively the resistance of the semi‐permeable membrane of the inserts. The resistances of the cell cultures were set in relation to this blank. Before using the device, it was desinfected with 70% ethanol for 15 min. Epithelial Voltometer (EVOM; World Precision Instruments, Sarasota, FL, USA) generated an alternating current at a frequency of 12.5 Hz. The EVOM was calibrated to 1000 𝛺, and after the 15 min of desinfection, the electrode was briefly rinsed in sterile H_2_0 and then adjusted to the pH of BEGM without additives for 15 min. Shortly before starting the measurements, 700 μL of BEGM was applied apically and 1300 μL basolaterally after removing the previous medium. Electrical resistance was measured at three positions of the membrane, and average values were calculated for each donor and exposure condition. Measured values were normalized to the cell culture area (1.12 cm^2^) to receive TEER in units of 𝛺xcm^2^.

### 2.5 Real‐Time Quantitative PCR (RT‐qPCR) Analysis

After 14 days of ALI culture, NEC were exposed to 0.1 ppm of NO_2_ for 1 h before gene expression of occludin as a key component of tight junctions was assessed. Furthermore, mRNA of IL‐6 und IL‐8 were measured after cell exposure to NO_2_ alone or under normoxic/hypoxic conditions. To extract RNA from cells, the Qiagen RNeasy Mini Kit (Qiagen GmbH, Hilden, Germany) was used according to the manufacturer's instructions. RNA concentration was determined spectrophotometrically (Eppendorf AG, Hamburg, Germany). cDNA was produced from 50 ng of RNA using 4 μL of Master Mix (SuperScript VILO Mastermix, Life Technologies Corp. [Applied Biosystems], Carlsbad, CA, USA) in Fast‐Reaction‐Tubes Micro Amp 8 Cap strips (Life Technologies Corp. (Applied Biosystems)]. cDNA was synthesized in the RT‐PCR device, StepOnePlus Real‐Time PCR System (Life Technologies Corp. (Applied Biosystems) and then mixed with the TaqMan Gene Expression Master Mix (Life Technologies Corp. (Applied Biosystems). The TaqMan Gene Expression Assay (Thermo Fisher Scientific, Waltham, MA, USA) was used with primers for Interleukin‐6 (IL‐6/Assay‐ID: Hs00985639_m1), Interleukin‐8 (IL‐8/ Hs00174103_m1), and Occludin (OCLN/ Hs05465837_g1). The internal housekeeping gene was Glyceraldehyde‐3‐phosphate dehydrogenase (GAPDH/Hs02758991_g1). Relative quantification of the results was determined with the ΔΔCT method.

#### 2.5.1 Statistical Analysis

The obtained data were statistically analyzed using the GraphPad Prism software 8 (GraphPad Software Inc., La Jolla, CA, USA). The D'Agostino–Pearson test and the Dunn's multiple comparison test (post hoc test) were used to statistically assess and evaluate the results in case of no normal distribution. When data were normally distributed, ANOVA and post hoc Tukey's multiple comparison test were performed. *p*‐values ≤ 0.05 were considered statistically significant.

## 3 Results

### 3.1 Changes in FITC‐Dextran Permeability Upon Transition to ALI Conditions

**FIGURE 1:**
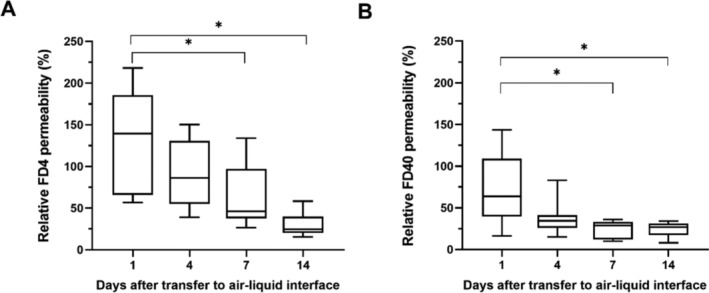
FD4 (A) and FD40 (B) passage was measured 1, 4, 7, and 14 days after the transfer of NEC to ALI culture conditions. Permeability decreased at each of the time points, reaching a significant difference compared to Day 1 after 7 days for both tested dextran sizes. The permeability was calculated by dividing the measured fluorescence of the sample and the fluorescence of the apically applied FITC‐medium mixture, minus the fluorescence value of the pure medium. Asterisks indicate *p* ≤ 0.05 (Dunn's multiple comparison test). Data are presented with box plots, margins of them illustrate the 25th and 75th percentiles. Whiskers indicate minimal and maximal values. *n* = 10.

After transfer of the cultures to ALI, the cells increasingly differentiate, resulting in the formation of cilia and the production of mucus. This is accompanied by an increase in the maintenance of the epithelial barrier. FITC‐Dextran permeability was assessed as a representative of the barrier integrity after 1‐, 4‐, 7‐, and 14‐day post ALI culture condition beginning. FD4 (Figure 1A) and FD40 (Figure 1B) permeability decreased at each of the time intervals with a significant result after 7 and 14 days compared to day 1.

### 3.2 Effects of NO_2_ Exposure

**FIGURE 2:**
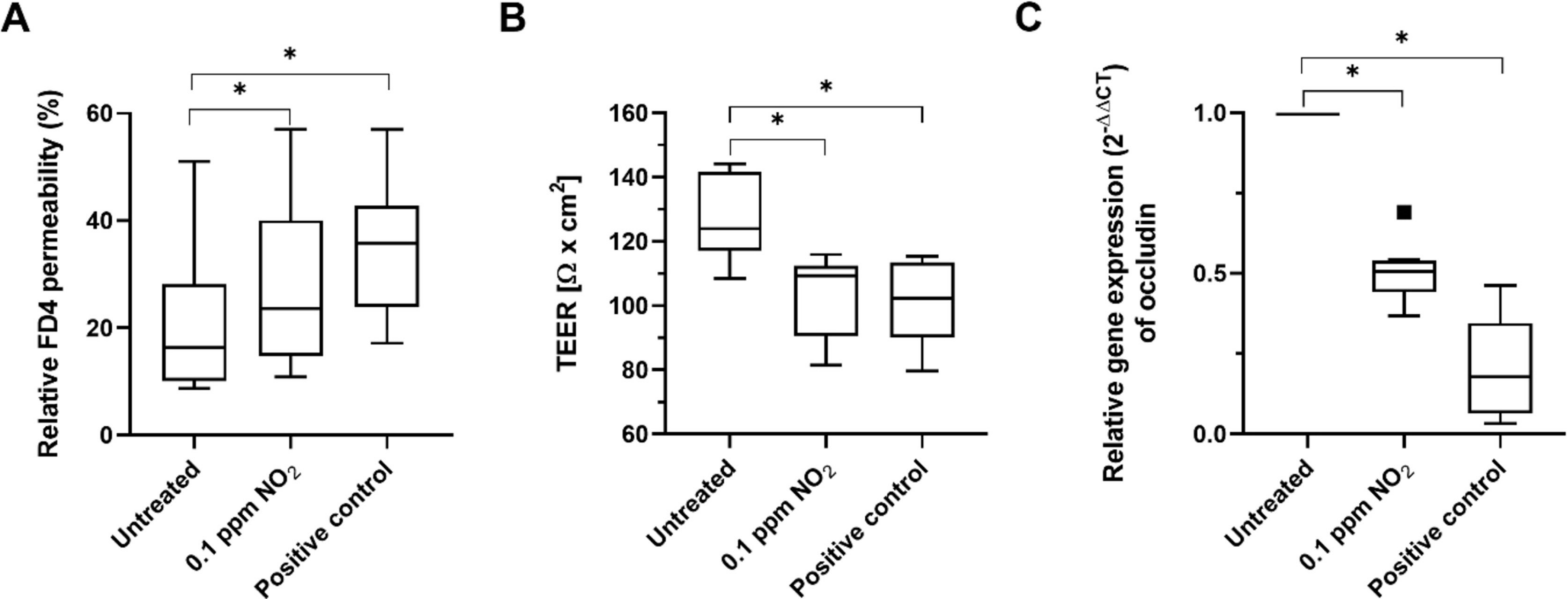
NEC were exposed to 0.1 ppm of NO_2_ for 1 h. LPS (for FITC‐dextran and RT‐PCR) and tBHP (for TEER measurement) served as positive controls. FD4 permeability measured by FITC‐dextran assay (Figure 2A) increased and TEER (Figure 2B) significantly decreased in treated cells compared to the untreated control. Figure 2C shows the result of the quantitative Real‐Time PCR of occludin. Data are presented as 2^‐ΔΔCT^, so that a change of 1 theoretically corresponds to a doubling of the number of mRNA. Untreated cells were set to 1. Asterisks indicate *p* ≤ 0.05 (as data were normally distributed, multiway ANOVA followed by Tukey's multiple comparison test was performed). The square in the boxplot in Figure 2C indicates a statistical outlier (outside 1.5 standard deviations from median); *n* = 10.

All the following experiments were conducted on cells cultured at the ALI for 14 days, a time point identified to have significantly higher barrier integrity compared to immediately after transfer to ALI. Donor‐specific variance is known to be high in primary cell‐based models, which is reflected in the variance of the data. Cells were exposed to 0.1 ppm of NO_2_ for 1 h before measuring FITC‐dextran permeability, TEER values, and isolating mRNA for gene expression analysis of occludin. Figure 2 demonstrates that FD4 permeability significantly increased and TEER decreased compared to untreated cells after NO_2_ exposure. Moreover, mRNA levels of occludin were significantly lower in treated cells compared to the untreated control.

### 3.3 Effects of Combined or Individual Exposure to NO_2_ and Hypoxia

**FIGURE 3:**
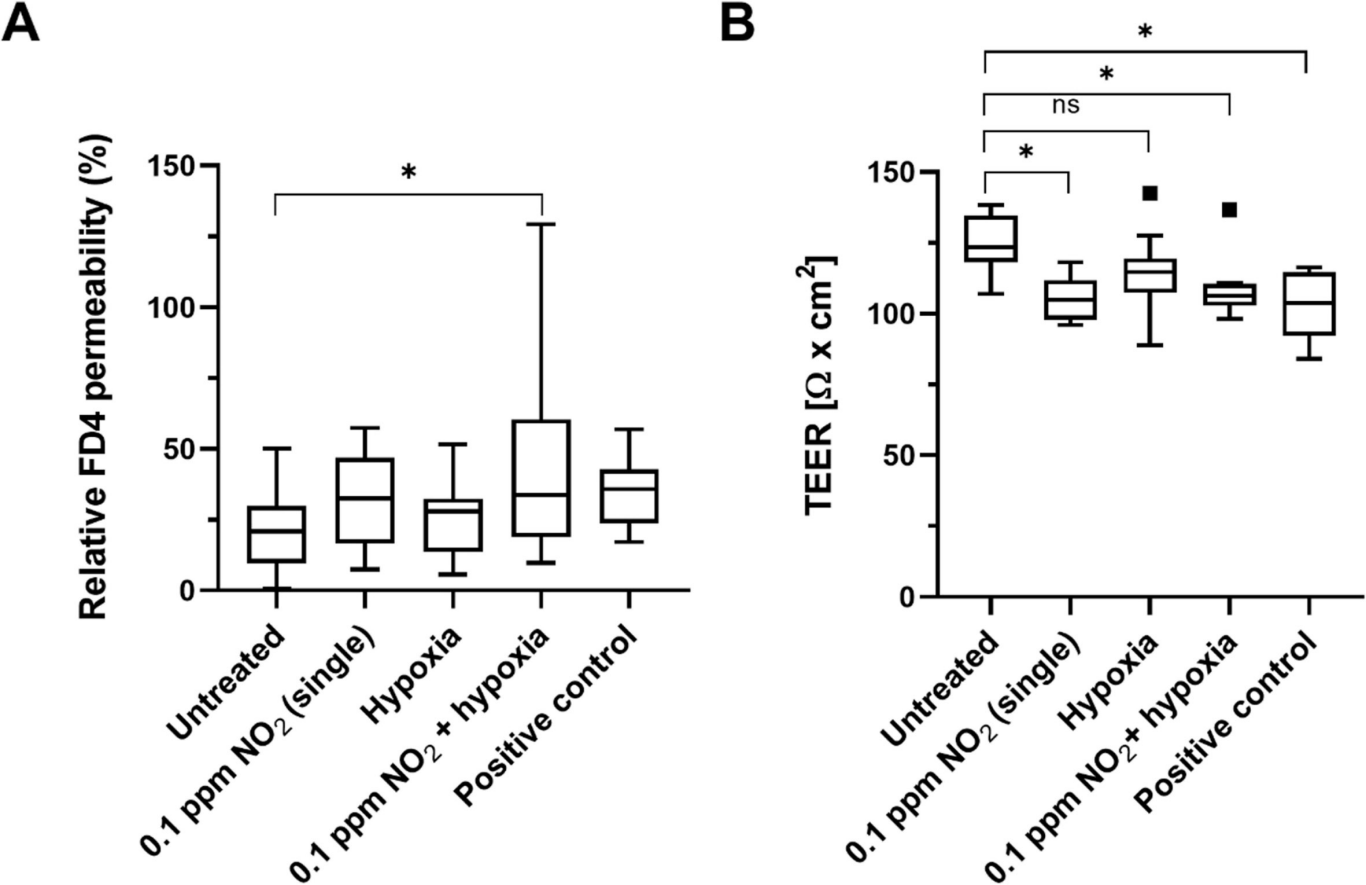
Results of FD4 permeability (A) and TEER measurement (B). NEC were exposed to 0.1 ppm of NO_2_ alone, hypoxia alone, or in combination (NO_2_ + hypoxia). Untreated cells served as the negative control, while cells treated with LPS for FITC‐dextran assay and tBHP for TEER analysis served as the positive control. There was a significant increase in FD4 passage after NO_2_ + hypoxia and decrease in TEER in the NEC exposed to NO_2_ alone and NO_2_ + hypoxia. Results were not significant for the hypoxia single exposure group compared to untreated cells. Asterisks indicate *p* ≤ 0.05 (as data were normally distributed, multiway ANOVA followed by Tukey's multiple comparison test was performed). Squares indicate statistical outliers (outside 1.5 standard deviations from median); *n* = 10.

Five test groups were established for this experiment: (1) untreated NEC as a negative control, which corresponds to incubation in normoxia; (2) positive control treated with LPS for FITC‐dextran and RT‐PCR, and tBHP for TEER measurement; and the test groups with (3) exposure to 0.1 ppm of NO_2_ exclusively; (4) 0.1 ppm of NO_2_ and hypoxia; and (5) sole exposure to hypoxia. Multiway ANOVA followed by Tukey's multiple comparison test revealed a significant increase in the basolateral FD4 concentration between the untreated cells and combined exposure with NO_2_ and hypoxia (*p* = 0.0161). The TEER measurement also showed a significant decrease in transepithelial resistance between the negative and positive control (*p* = 0.002), as well as between the negative control and sole NO_2_ exposure (*p* = 0.0012) and combined exposure with NO_2_ and hypoxia (*p* = 0.0081). The comparison of negative control and hypoxia alone was not significant in either test. Moreover, there was no significant difference between NO_2_ exposure alone and NO_2_ + hypoxia. Figure 3 demonstrates the results for the FITC‐dextran assay (A) and TEER measurement (B).

### 3.4 mRNA Expression of IL‐6 and IL‐8

**FIGURE 4:**
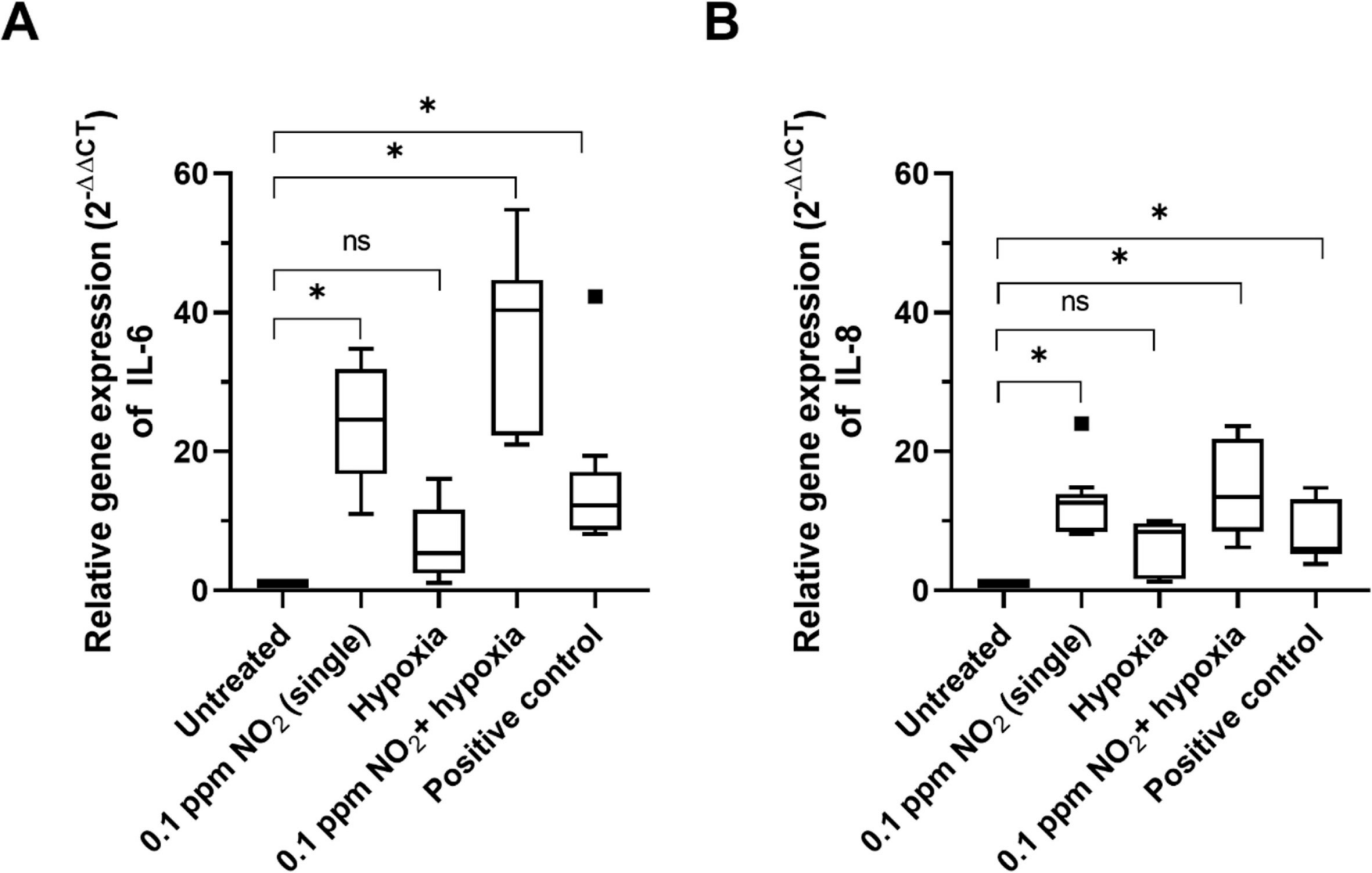
IL‐6 (A) and IL‐8 (B) mRNA abundance determined by qRT‐PCR. NEC were exposed to 0.1 ppm of NO_2_ alone, hypoxia alone, or in combination (NO_2_ + hypoxia). LPS was used as positive control. Data are presented as 2^‐ΔΔCT^, with untreated cells set to 1. IL‐6 was significantly elevated after NO_2_ exposure alone and in combination with hypoxia. Hypoxia alone did not induce significant changes. IL‐8 levels were significantly altered in all subgroups compared to the negative control. Asterisks indicate *p* ≤ 0.05 (as data were normally distributed, multiway ANOVA followed by Tukey's multiple comparison test was performed). Squares indicate statistical outliers (outside 1.5 standard deviations from median); *n* = 10.

qRT‐PCR was performed to analyze mRNA levels of IL‐6 and IL‐8 in NEC after exposure to 0.1 ppm of NO_2_, hypoxia alone, or in combination with both. As shown in Figure 4, there was significantly more IL‐6 and IL‐8 mRNA compared to the negative control in all groups except the hypoxia single exposure subset. Treatment with NO_2_ + hypoxia induced significantly stronger IL‐6 expression compared to treatment with NO_2_ alone. However, the difference in IL‐8 expression between NO_2_ + hypoxia and NO_2_ alone was not significant.

## 4 Discussion

NO_2_ is a ubiquitous gaseous air pollutant, and given its harmful effects on health, accurate toxicological characterization is important. Prior research has predominantly centered on examining effects on the lower airways, despite the fact that the upper airways serve as the initial point of contact with inhaled substances such as NO_2_. Therefore, the present study was designed with the aim to investigate functional effects on NECs caused by NO_2_ and elucidating their molecular mechanisms related to early inflammatory responses. Based on the recommendations of the World Health Organization (WHO), the 1‐h maximum exposure threshold of NO_2_ should not exceed 200 μg/m^3^, which equates to 0.1 ppm. This threshold should not be surpassed more than 18 times per year. The conditions selected for this study aim to provide insights into the effects of NO_2_ under realistic exposure scenarios and contribute to the assessment and interpretation of the current limit value. However, it is important to note that the study conditions simulate a short‐term, high‐level NO_2_ exposure, which does not perfectly reflect the more varied and heterogeneous exposure patterns typically encountered in real‐world settings Lancet (2006).

ALI‐based cultures from primary nasal mucosa cells were used as cellular model, which enabled a good in vitro–in vivo correlation in terms of functionality. ALI cultures are a suitable approach for mimicking the biology of airway epithelia, as evidenced by their transcriptional profile closely mirroring that of the in vivo situation. On the one hand, primary cells offer the advantage of closely resembling the original tissue in terms of cellular composition, histological features, and function, rendering them biologically relevant and reflective of certain in vivo conditions. Rodenburg et al. showed that the RNA profile is preserved in human nasal and bronchial ALI primary cell models (Rodenburg et al. 2023). This confirms that ALI models effectively reflect the characteristics of the tissue, at least at the RNA level. This allows for a more authentic response to pharmacological agents and toxic substances. On the other hand, primary cells are limited in availability and inherently exhibit high donor variability (Pezzulo et al. 2011; Upadhyay and Palmberg 2018). As support of an intact epithelial barrier, the first step was a consecutive temporal analysis of FITC‐dextran permeability after transferring the cultures into the ALI system. After 7 days, the permeability had significantly decreased in relation to the initial value, with further improvement observed until Day 14. For the subsequent experiments, the time point was therefore set at Day 14 after the start of ALI. In other in vitro models of airway epithelia, cultures are often maintained in ALI for 21 days before exposure experiments are conducted (Cozens et al. 2018; H. Wang et al. 2018). Furthermore, regular assessment of TEER would be an additional and valuable tool for verifying the integrity of the epithelial barrier. The models used in this study represent a simplified version, and models with different cellular components can certainly offer more realistic conditions. However, within our experimental setup, the cell models provided consistent results, suggesting their biological relevance.

While research data on NO_2_‐associated effects in primary human bronchial epithelial cells exist (Mirowsky, Dailey, and Devlin 2016; Upadhyay et al. 2022), there are few studies to focus on nasal mucosa cells as the subject of investigation. In this context, Schierhorn et al. demonstrated that NO_2_ and O_3_ trigger an inflammatory response in human nasal mucosa in vitro. Furthermore, they found that predisposing factors such as atopy or preexisting inflammation heighten the sensitivity to ozone and NO_2_ (Schierhorn et al. 1999). Hypoxia has been discussed as a pathophysiological factor in the development and maintenance of CRS. In individuals with CRS, the chronic inflammation of the nasal cavity can induce hypoxia in nasal mucosal tissues. This condition is worsened by sinus obstruction and mucosal swelling, thereby initiating a self‐perpetuating cycle (Zhong et al. 2022). It has been shown that intermittent hypoxia impairs mucociliary transport and elevates the expression of inflammatory factors like IL‐6, IL‐8, transforming growth factor‐β (TGF‐β), granulocyte‐macrophage colony‐stimulating factor, and TNF‐α in human nasal mucosa (In et al. 2021). Accordingly, an additive effect caused by hypoxia and NO_2_ is conceivable. To assess the induction of inflammatory mechanisms, the expressions of the factors IL‐6 and IL‐8 were measured. There is evidence suggesting that even systemic inflammation, characterized by increased serum IL‐6 levels, is present in individuals exposed to NO_2_ (Perret et al. 2017).

In this study, cell treatment with NO_2_ induced responses in epithelial cells, manifested by the decrease in TEER values and the increase in FITC‐dextran permeability. No alteration in epithelial barrier integrity due to hypoxic conditions could be observed. Furthermore, combined exposure to NO_2_ and hypoxia did not result in an additive effect with greater barrier damage compared to NO_2_ alone. The results are in contrast to the findings of Wong et al., who demonstrated a decrease in TEER levels under hypoxic conditions in nasal mucosa donors, both with and without cystic fibrosis (Wong et al. 2023). It is possible that the time of incubation in hypoxic conditions of 4 h was not sufficient to manifest functional epithelial damage in this study. Occludin, a central protein of the AJC, was selected as a target structure to examine alterations in the composition of cell–cell contacts. A reduced expression of occludin could indicate a beginning epithelial barrier affection after NO_2_ exposure. A connection between long‐term NO_2_ exposure and changes in tight junction (TJ) integrity in bronchioles and alveoli has already been described in a mouse model (Gordon, Solano, and Kleinerman 1986). Varied expression patterns of TJ molecules have been observed in different inflammatory phenotypes of allergic airway inflammation in mice compared to control groups (Tan et al. 2019). These results imply that the disruption of TJ may contribute to the onset of allergies by enhancing the exposure of nasal tissues to environmental allergens or other toxic substances. For instance, it has been demonstrated that decreased occludin expression is linked to urban environments and exposure to second‐hand smoke among individuals with AR (Nur Husna et al. 2021). Occludin is commonly used as a marker for studying epithelial barrier integrity, as demonstrated in different ALI‐based studies (Bigot et al. 2022; Park et al. 2024; Xian et al. 2020). Broadening the target proteins to include other components of cell–cell contacts, such as claudin or E‐cadherin, could enhance the significance of the research. The expression of occludin is highly variable and influenced by a combination of individual, cell‐specific, and dynamic factors. Its expression and localization are not static but are subject to rapid modulation in response to various external stimuli. For instance, it has been shown that occludin expression can change significantly within just 1 h of cold exposure in cells (Zhou et al. 2023). In the context of a pro‐inflammatory response, which is assumed to be an important factor after NO_2_ exposure, cytokines are likely to play a pivotal role in altering the mRNA expression of occludin. Additionally, oxidative stress may also contribute to these changes, warranting further investigation in future studies (Al‐Sadi, Boivin, and Ma 2009). LPS is a well‐established positive control in experiments assessing barrier integrity. It has been demonstrated that after 24 h of exposure, LPS leads to the disintegration of key tight junction components, such as zonula occludens‐1 (ZO‐1), resulting in a subsequent increase in paracellular permeability. However, it is important to note that LPS has also been reported to cause an upregulation of occludin (Kim et al. 2021). This highlights the dynamic nature of TJ component composition and underscores the need for analysis at the protein level to enhance the significance of the findings. Techniques such as immunoblotting or confocal imaging can be employed for this purpose. IL‐6 and IL‐8 expressions were measured as indicators for an inflammatory response to NO_2_ and hypoxia. The findings indicate a significant rise in IL‐6 and IL‐8 gene expression following exposure to NO_2_, while hypoxia did not elicit any change compared to untreated samples. However, the combined treatment of NO_2_ + hypoxia resulted in a significantly greater increase in expression compared to NO_2_ alone. In a review, Hesterberg et al. postulated that overall, there is inconclusive and inconsistent evidence for markers of pulmonary inflammation from short‐term NO_2_ inhalation exposures at concentrations below 1 ppm among healthy volunteers. The evidence becomes more consistent at higher NO_2_ concentrations, typically between 1.5 and 2 ppm (Hesterberg et al. 2009). In contrast, the present study demonstrates that exposure to 0.1 ppm of NO_2_ induces the expression of inflammatory mediators at the mRNA level. However, it is important to note that the measurement was limited to qRT‐PCR analysis at the mRNA level, and therefore, no conclusions can be drawn regarding resulting protein expression. Assessing the impact of inflammation on the organism is challenging, and qRT‐PCR analysis was chosen as it can detect changes in mRNA expression that precede changes at the protein level, providing early insights into cellular responses. The short half‐lives of IL‐6 and IL‐8 make it difficult to use protein analysis to determine these parameters, which complicates the use in a primary cell model.

In conclusion, our findings add information on harmful effects of NO_2_ exposure on the nasal epithelium, including compromised barrier integrity and induction of inflammatory responses. This observation may be related to a dysregulation of TJ components, indicated by a reduced mRNA expression of occludin. This work provides insights into the effects of NO_2_ on the epithelium of the upper respiratory tract, an area with currently still limited information. The findings contribute to our understanding of the pathophysiological mechanisms underlying respiratory diseases associated with air pollution exposure and underscore the importance of mitigating NO_2_ emissions to protect respiratory health.

Future studies should incorporate 3D culture models to create more realistic study conditions. Additionally, considering previous damage to the respiratory nasal epithelium from other environmental pollutants or cigarette smoke will be crucial in understanding individual susceptibility to NO_2_. This comprehensive approach will further elucidate the complex interactions between environmental factors and respiratory health, guiding more effective public health interventions.

## Ethics Statement

The study was conducted according to the guidelines of the Declaration of Helsinki and approved by the Medical Department Ethics Board of the Julius Maximilians University Wuerzburg (116/17).

## Consent

Informed consent was obtained from all subjects involved in the study.

## Conflicts of Interest

The authors declare no conflicts of interest.

## Data Availability

The data that support the findings of this study are available from the corresponding author upon reasonable request.
